# Combined Toxicity of Cannabidiol Oil with Three Bio-Pesticides against Adults of *Sitophilus Zeamais*, *Rhyzopertha Dominica*, *Prostephanus Truncatus* and *Trogoderma Granarium*

**DOI:** 10.3390/ijerph17186664

**Published:** 2020-09-13

**Authors:** Spiridon Mantzoukas, Nikolaos Kalyvas, Aristeidis Ntoukas, Ioannis Lagogiannis, Konstantinos Farsalinos, Panagiotis A. Eliopoulos, Konstantinos Poulas

**Affiliations:** 1 Department of Pharmacy, University of Patras, 26504 Patras, Greece; nikolaoskalyvas3@gmail.com (N.K.); ntoukasaris7@gmail.com (A.N.); lagoipp@gmail.com (I.L.); kfarsalinos@gmail.com (K.F.); 2 Department of Agrotechnology, University of Thessaly, Gaiopolis, 41500 Larissa, Greece; eliopoulos@uth.gr

**Keywords:** CBD oil, biopesticides, stored pests, insecticidal, Azatin, madex, helicovex

## Abstract

The present study investigates the interaction between cannabidiol (CBD) oil and three biopesticides: Azatin and two baculovirus formulations (Madex and Helicovex), both separately and in combination, in order to investigate their interaction against adults of four major coleopteran stored-product pests: *Sitophilus zeamais* (Coleoptera: Curculionidae), *Rhyzopertha dominica* (Coleoptera: Bostrichidae), *Prostephanus truncatus* (Coleoptera: Bostrichidae) and *Trogoderma granarium* (Coleoptera: Dermestidae). CBD, which has been understudied for its insecticidal properties, was applied at three different doses (500, 1500 and 3000 ppm). The biopesticides were administered at 1500 ppm. Interactions in the combined treatments were mathematically estimated as not synergistic and mostly competitive except for the combined treatments of CBD (1500 and 3000 ppm) with Azatin (1500 ppm) which were marked by an additive interaction. In its individual application, CBD oil generated the highest insect mortality while its effect was clearly dose-dependent. The findings reveal a promising effect of CBD oil against these coleopterans which had not been previously tested together.

## 1. Introduction

Insect pests cause severe damage to plant life and crops with an estimation of approximately 30% of crop yields lost due to pathogens [1], rendering their control a crucial issue for farmers worldwide. Although chemical pesticides remain the most effective method for the management of serious storage pests, their action has proven to be hazardous for the user and the environment [2,3]. Moreover, their extensive and often unnecessary application has increased resistance to chemical pesticides in target species [4]. Appreciation for the health and environmental risks involved in the use of chemical pesticides has propelled a shift towards the search for biopesticides as well as botanicals with insecticidal and repellent effects. Botanical insecticides present an array of advantages including low mammalian toxicity, environmental safety, efficacy as well as a reduced likelihood to cause resistance in arthropods [5,6,7], and as such, they have legitimately attracted a high degree of interest for their pest control potential, including in the management of stored-product pests [8].

The use of entomopathogenic microorganisms is central in biological pest control [9,10,11,12,13,14]. Entomopathogenic viruses are among the microorganisms whose value has been acknowledged in biological pest control as they exhibit good insecticidal potential, high selectivity, making them safe for nontarget arthropods, and compatible with other pest control methods [15]. *Cydia pomonella* Granulovirus (CpGV) and *Helicoverpa armigera* Nucleopolyhedrovirus (HearNPV) are two baculoviruses with proven efficacy against Lepidopteran pests. Research has shown that HearNPV can be utilized as a control agent for pests which attack chickpea and sunflower crops [16,17]. Likewise, many plants can be used as biopesticides, as many plant species are famous for their insecticidal properties [18,19,20,21]. A widely used plant species is *Azadirachta indica* A. Juss (Sapindales: Meliaceae). Numerous studies highlight the unique properties of its extracted oil and its effectiveness as an insecticide [22,23,24]. Research has also been conducted over recent years on *Cannabis sativa* L. (Rosids: Cannabaceae) and, especially, one major cannabinoid that is present in the plant which is cannabidiol (CBD). Cannabis-based insecticides and repellents are widely available [5,25]; however, the mode of action of CBD as an insecticide or repellent is still under investigation.

Two or more biopesticides are often combined to create an enhanced product with a better efficiency or duration [26,27,28]. However, they may not coexist harmoniously in a mixture and their action is not always additive. Their interaction can be synergistic or antagonistic [29,30,31,32,33,34,35], depending on the concentration, species, mode of action, target organism, temperature conditions, and relative humidity (r.h) conditions of each biopesticide [33,34,35,36,37,38,39,40,41,42,43,44,45,46,47]. The combined use of two or more biopesticides in one mixture may result in less (or more) environmental harm or less (or more) harm to nontarget organisms [48,49,50]. Biopesticides based on plant essential oils and microorganisms are considered relatively safe and their efficacy has been demonstrated against different insect species.

The aim of the present study was to investigate the insecticidal effect of CBD oil in combination with three biopesticides: CBD + Madex (*Cydia Pomonella* Granulovirus (CpGV)), CBD + Azatin (Azadirachtin), and CBD + Helicovex (*Helicoverpa Armigera* Nucleopoyhedrovirus (HearNPV)). The above combinations were tested against adults of *Sitophilus zeamais* (Coleoptera: Curculionidae), *Rhyzopertha dominica* (Coleoptera: Bostrichidae), *Prostephanus truncatus* (Coleoptera: Bostrichidae), and *Trogoderma granarium* (Coleoptera: Dermestidae). This study is a follow up on previous similar studies investigating the synergism between biopesticides, and biopesticides and botanicals to enhance toxicity against stored-product pests [26,27,28]. CBD oil and these three biopesticides have never been investigated for their combined action against the four coleopteran pests. This is the first time CBD oil has been tested against the specific coleopteran pests.

## 2. Materials and Methods

### 2.1. Insect Rearing

Four major stored-product beetle species were selected: the maize weevil *S. zeamais*, the lesser grain borer *R. dominica*, the larger grain borer *P. truncatus* and the khapra beetle, *T. granarium*. These species cover a wide spectrum of globally distributed stored-product pests and cause serious losses and degradations to a wide range of commodities. All species were reared in a growth chamber (PHC Europe/Sanyo/Panasonic Biomedical MLR-352-PE) in controlled environmental conditions, at 27.5 °C and 75% relative humidity (r.h.) in the Laboratory of Molecular Biology and Immunology, Department of Pharmacy, University of Patras. We used mixed sex adults, <2 weeks old. The bioassays were carried out on wheat (var. Mexa). This grain was adjusted at 12% moisture content (m.c.), via storage in ambient conditions, for 28 d. Whole grain kernels were used because these species are known to infest sound grains.

### 2.2. Bioassay

Individual lots of 500 g of wheat were placed in 0.45 L cylindrical glass jars, in the laminar flow cabinet (Equip Vertical Air Laminar Flow Cabinet Clean Bench. Mechanical Application LTD. Athens. Greece). The CBD solution (3% oil produced by Enecta Athens, Greece) was diluted with methanol (MeOH) and was applied on the commodities at three concentrations (500, 1500 and 3000 ppm). The same process was followed for the biopesticides whereby Madex (*Cydia pomonella* Granulovirus (CpGV)) (Hellafarm, Athens, Greece), Azatin (Azadirachtin A 2.6 EC) (K&NE Earth Matters, Thessaloniki, Greece), and Helicovex (*Helicoverpa αrmigera* Nucleopoyhedrovirus (HearNPV)) (Hellafarm, Athens, Greece) were tested at 1500 ppm. Solutions were sprayed on the products using the Potter spray tower (Burkard Manufacturing Co. Ltd., Rickmansworth, Hertfordshire, U.K.) at 1 kgf cm^−2^. The entire process was repeated twenty times, whereby new groups of insects were prepared each time. After the solutions had been sprayed on the product, the wheat lots were placed back into the jars and were shaken manually for 30 s to achieve an equal distribution of the treatment. The product was then air dried for 30 min especially for the CBD oil concentrations because methanol evaporates within a short time. Separate lots of wheat were sprayed with distilled water and MeOH, to serve as controls. From each jar, ten 20 g samples were taken and placed in cylindrical plastic vials (3 cm in diameter, 8 cm in height). The vials had a plastic lid with a hole in the center covered with fine mesh, while the internal “neck” of the vials was covered with Fluon (Northern Products, Woonsocket, RI USA), to prevent adult insects from escaping. Twenty individuals were placed within each vial (separate vials for each species) and the vials were placed in plastic boxes with saturated solutions of sodium chloride to maintain 75% r.h. Two hundred coleopteran adults were used for each concentration (20 adults per replication) and the experiment was replicated ten times. All boxes were then placed in incubators set at 27.5 °C and 75% r.h. Adults were observed daily, and mortality was recorded at 7, 14, 21 and 28 days after treatment.

To study the combined effect of the treatments, adult insects were treated with a combination of each biopesticide with a CBD oil suspension, resulting in nine different combinations (Azatin 1500 ppm + CBD 500 ppm, Azatin 1500 ppm + CBD 1500 ppm, Azatin 1500 ppm + CBD 3000 ppm; Madex 1500 ppm + CBD 500 ppm, Madex 1500 ppm + CBD 1500 ppm, Madex 1500 ppm + CBD 3000 ppm; Helicovex 1500 ppm + CBD 500 ppm, Helicovex 1500 ppm + CBD 1500 ppm, Helicovex 1500 ppm + CBD 3000 ppm). The product was initially sprayed with 2 mL of the CBD oil and, after 2 s, with 2 mL of the biopesticide concentration. Twenty individuals were placed within each vial (separate vials for each species), and the vials were placed in plastic boxes with saturated solutions of sodium chloride to maintain 75% r.h. Replications and spray procedures for the combined treatments were carried out as in the individual treatments. Adults were observed daily, and mortality was recorded at 7, 14, 21 and 28 days after treatment.

### 2.3. Mathematical Estimation

The interaction between the pathogens was estimated using the formula of Robertson and Preisler [51]:

*P*_E_ = *P*_0_ + (1 − *P*_0_) × (*P*_1_) + (1 − *P*_0_) × (1 − *P*_1_) × (*P*_2_) (1)

where *P*_E_ is the expected mortality induced by the combination of the two pathogens; *P*_0_ is the observed mortality of the control; *P*_1_ is the observed mortality caused by the first pathogen (separate action); *P*_2_ is the observed mortality caused by the second pathogen (separate action). The distribution was determined by the chi-square formula:

x^2^ = (*L*_0_ − *L*_E_)^2^/*L*_E_ + (*D*_0_ − *D*_E_)^2^/*D*_E_ (2)

where *L*_0_ is the number of recorded live larvae of the control. *D*_0_ is the number of recorded dead larvae of the control, *L*_E_ is the expected number of live larvae, and *D*_E_ is the expected number of dead larvae (estimated the same as *P*_E_, with Equation (1)). The formula was used to test the hypothesis independent–simultaneous relationship (*d*_f_ = 1, *p* = 0.05). If x^2^ < 3.84, the ratio is defined as additive, if x^2^ > 3.84 and the observed mortality is higher than expected, the relationship is defined as synergistic. On the contrary, if x^2^ > 3.84 and the observed mortality is less than expected, the relationship is defined as competitive.

### 2.4. Statistical Analysis

All values were arcsine transformed prior to the analysis to reduce the effect of the variance. Data were analyzed by a two-way ANOVA using the general linear model of the SPSS (version 25) (IBM 2019, Armonk, NY, USA). In case of significant F values, means were compared using the Bonferroni test. The Kaplan–Meier method of the SPSS (ver. 25) was also selected to determine the mean survival time of *S. zeamais*, *R. dominica*, *P. truncatus* and *T. granarium* adults following the application of the treatments. The Cox regression of the SPSS (ver. 25), a common survival analysis regression method that describes the relation between the event incidence and a set of covariates, was selected to determine the hazard effect of the individual and combined treatments.

## 3. Results

Essential oil and biopesticides induced significantly different levels of mortality on adults of *S. zeamais*, *R. dominica*, *P. truncatus* and *T. granarium*. Significant differences were recorded between treatment (F = 108.272, *d*_f_ = 6.139, *p* < 0.001) and replication (F = 10.909, *d*_f_ = 4.139, *p* < 0.001) but not between the coleopteran species of the experiment (F = 1.911, *d*_f_ = 3.139, *p* = 0.132) as factors, in relation to the dependent variable of mortality at 28 days. The two-way factor model of coleopteran species × treatment (F = 0.9722, *d*_f_ = 18.139, *p* = 0.001) and treatment × replication (F = 4.950, *d*_f_ = 24.139, *p* = 0.001) showed a significant effect in terms of the mortality of coleopteran adults at 28 days. The mean beetle mortality caused by the separate action of CBD Oil, Azatin, Helicovex and Madex is presented in Table 1. More specifically, 28 days after the treatment with CBD oil, the mortality of *S. zeamais* adults increased from 38% (500 ppm) to 70% (3000 ppm), of *R. dominica* from 44% (500 ppm) to 84% (3000 ppm), of *P. truncatus* from 48% (500 ppm) to 80% (3000 ppm), and of *T. granarium* from 48% (500 ppm) to 62% (3000 ppm) (Table 1). At 28 days, Azatin-induced mortality to Coleoptera was between 38% (*T. granarium*) and 50% (*P. truncatus*); Helicovex-induced mortality was between 16% *(S. zeamais*) and 20% (all others) (Table 1); Madex-induced mortality was between 18% (*S. zeamais*) and 22% (*R. dominica*) (Table 1).

A total of nine combined treatments of CBD oil with the biopesticides were applied against *S. zeamais. R. dominica. P. truncatus* and *T. granarium*. Significant differences were recorded between treatment (F = 57.641, *d_f_* = 8.179, *p* < 0.001) and between the tested insects of the experiment (F = 14.149, *d*_f_ = 3.179, *p* < 0.001) as factors, in relation to the dependent variable of mortality, at 28 days. The adult mortality of the four beetles varied significantly in terms of the combinations at two-way factor insect * treatment (F = 2.138, *d*_f_ = 24. 179, *p* = 0.003) at 28 days. After 28 days, the mean mortality of the combined treatments of CBD Oil, Azatin, Helicovex and Madex was, for *S. zeamais* adults treated with CBD and Azatin between 36% (CBD 500–Azatin 1500 ppm) and 84% (CBD 3000–Azatin 1500 ppm), with CBD and Helicovex between 30% (CBD 500–Helicovex 1500 ppm) and 62% (CBD 3000–Helicovex 1500 ppm), and with CBD and Madex between 18% (CBD 500–Madex 1500 ppm) and 40% (CBD 3000–Madex 1500 ppm) (Table 2); for *R. dominica* adults treated with CBD and Azatin, the mean mortality was between 50% (CBD 500–Azatin 1500 ppm) and 90% (CBD 3000–Azatin 1500 ppm), with CBD and Helicovex between 40% (CBD 500–Helicovex 1500 ppm) and 70% (CBD 3000–Helicovex 1500 ppm), and with CBD and Madex between 32% (CBD 500–Madex 1500 ppm) and 62% (CBD 3000–Madex 1500 ppm) (Table 2); for *P. truncatus* adults treated with CBD and Azatin, the mean mortality was between 44% (CBD 500–Azatin 1500 ppm) and 94% (CBD 3000–Azatin 1500 ppm), with CBD and Helicovex between 38% (CBD 500–Helicovex 1500 ppm) and 70% (CBD 3000–Helicovex 1500 ppm), and with CBD and Madex between 26% (CBD 500–Madex 1500 ppm) and 52% (CBD 3000–Madex 1500 ppm) (Table 2); for *T. granarium* adults treated with CBD and Azatin, the mean mortality was between 50% (CBD 500–Azatin 1500 ppm) and 62% (CBD 3000–Azatin 1500 ppm), with CBD and Helicovex between 46% (CBD 500–Helicovex 1500 ppm) and 68% (CBD 3000–Helicovex 1500 ppm), and with CBD and Madex between 38% (CBD 500–Madex 1500 ppm) and 54% (CBD 3000–Madex 1500 ppm) (Table 2).

The results of the combined treatments showed different types of interaction between the biopesticides and CBD oil. In the cases of *S. zeamais*, the interaction between the treatments was competitive except for one (Table 3). For *P. truncatus*, the interaction between the treatments was competitive except two, and for *R dominica*, the interaction between the treatments was competitive except three (Table 3). An additive interaction was recorded for the combined treatments of the CBD oil (1500 and 3000 ppm) with Azatin (1500 ppm) for all above beetles (Table 3). By contrast, Helicovex and CBD oil marked an additive interaction against *T. granarium* at all three doses, and against *R. dominica* at one dose (Table 3). No synergistic relationship was recorded in any of the combinations (Table 3).

The six combined treatments which were overall toxic to *S. zeamais*, *R. dominica*, *P. truncatus* and *T. granarium* adults had positive β-values (Table 4). The combined treatments which exhibited a dose-dependent action were CBD 3000–Madex 1500 ppm, CBD 1500–Helicovex 1500 ppm, CBD 3000–Helicovex 1500 ppm, CBD 500–Azatin 1500 ppm, CBD 1500–Azatin 1500 ppm, and CBD 3000–Azatin 1500 ppm. The overall Hazard Rate (Exp (B)) for all combined treatments was higher than the control. Finally, the above treatments were not statistically significant, except for CBD 3000–Helicovex 1500 ppm, CBD 1500–Azatin 1500 ppm and CBD 3000–Azatin 1500 ppm with *p* values of <0.001, <0.001 and <0.001, respectively (Table 4). The individual treatments which also had an effect on the overall mortality of *S. zeamais*, *R dominica*, *P. truncatus* and *T. granarium* adults had positive β-values and included all three CBD oil concentrations; they were also statistically significant from the remaining individual treatments. The overall hazard rate (Exp (B)) for the CBD oil concentrations was higher than the control.

## 4. Discussion

The present study represents an investigation of the individual and combined insecticidal effect of CBD oil and three biopesticides, Azatin, Madex and Helicovex. In our study, the combined treatments of CBD with the biopesticides produced varying results. Our hypothesis was that the interaction between treatments would be more additive than synergistic. Insects would thus die from a reinforced action. Our results support this hypothesis in the case of CBD with Azatin for all tested insects, as well as in the case of two insects with CBD and Helicovex. Overall, no combination exhibited synergism.

On its own, CBD oil yielded the highest mortality rates at 28 days. Its action was dose-dependent which is generally the case with essential oils, whose effects include metabolic and physiological disruptions, neurotoxic effects, and histological changes [29]. Benelli et al. [5] report that the essential oil from the inflorescence of industrial hemp was highly toxic to aphids and flies, although results ranged from moderate to scarcely toxicity larvae and adults. *C. sativa* L and CBD-related studies on their insecticidal properties are scarce. In most studies, *C. sativa* was tested as a companion plant, harvested plant material, in aqueous and solvent extracts, and as essential oil [25]. There is bound to be variation in the findings concerning the insecticidal effects of the plant. In their review, McPartland and Sheikh [25] concluded that of all bioassays, the essential oil produced the best results in the treated insects which were mainly thin-cuticle small arthropods.

Azadin (1500 ppm) generated moderate to low mortality reaching a peak at 50% (*P. truncatus*) [30]. Similarly, albeit as a crude powder, *A. indica* was tested against *P. truncatus* and was found to be weakly toxic to the insect, causing a mere mortality of 40% at 28 days [30]. In a similar vein, azadirachtin-enriched neem kernel extracts on wheat consumed by *R. dominica* did not have any effect on parent mortality although they inhibited F_1_ progeny by 98% [47]. Tofel et al. [48] found Azadirachtin seed oil to be lethal to *Callosobruschus maculatus* Fabricius (Coleoptera: Chrysomelidae) and *S. zeamais* adults. Variation in the effect of azadirachtin and neem extracts may be the product of differences in the derivatives and formulations used as well as of a complex of several other factors such as grain type, target species, dosage, temperature, humidity and others [49]. In the present study, moderate to low mortality could be interpreted as an indirect event resulting from the disruption in the feeding of the insect, drawing on Xie et al. [51] in their interpretation of the killing of *Sitophilus oryzae* Linnaeus (Coleoptera: Curculionidae)*, Tribolium castaneum* Herbst (Coleoptera: Tenebrionidae) and *Cryptolestes ferrugineus* Ganglbauer (Coleoptera: Laemophloeidae).

Finally, Madex and Helicovex were ineffective as mortality rates did not exceed 22% at 28 days. The low mortality can be accounted for by the age of the insects, as viruses are mostly pathogenic to larvae whereby molting and pupation is blocked by the infection [52], and by the different insect order as the two baculoviruses are mostly effective against Lepidopterans. Moreover, they were slow in their action, which is a known disadvantage of viruses in general [53].

Examining how pesticides interact with each other advances research on to the creation of more effective, cost efficient and lasting pesticidal products. Interaction is determined by the interplay of many factors such as the compound itself, dose, insect species, and others. One or all individual components of a combination may enhance (synergism) or suppress (antagonism/competition) each other’s toxicity, or simply remain within the additive framework [54,55,56,57]. The observed additive effect with high toxicity values (B and Exp (B)) is the result of the Azadirachtin making insects more sensitive to CBD, thus adding to the result.

It is also worth pointing out that *T. granarium* (all concentrations) and *R. dominica* (CBD 1500–Helicovex 1500 ppm) were the only beetles in which CBD and the Helicovex did not compete, whereas CBD had a negative interaction with Madex or Helicovex in the case of the other two Coleoptera. Thus, an additive effect with very good toxicity values (B and Exp (B)) was shown when adults of *T. granarium* and *R. dominica* were treated with combined concentrations. The *T. granarium* and *R. dominica* cases are a finding worth exploring further.

On the contrary, the mathematical estimation revealed that most combinations were competitive. A competitive interaction refers to the negative relationship between pathogens. The nature of competition between tested treatments is not documented. In our case, the combined use of CBD with Madex exhibited negative B values but good toxicity values. The combined concentration of CBD with Madex did not produce increased hazard and decreased survival time in terms of the mortality of the beetles.

## 5. Conclusions

Although acute toxicity of plant-derived substances is lower than that of their chemical counterparts, they have attracted a high degree of interest for their pest control potential, including for the management of stored-product pests. As research in biological control is gaining considerable momentum, and as the list of entomopathogenic properties of plants and biopesticides is continually enriched, our findings highlight the potential of CBD against major stored-product pests and add to the limited literature concerning this essential oil.

## Figures and Tables

**Table 1:** Mean (%) mortality and median survival time (days) of adults of *S. zeamais*, *R. dominica*, *P. truncatus* and *T. granarium* treated separately with cannabidiol (CBD) oil, Azatin, Helicovex and Madex after 28 days. Mean% mortality for different concentrations or biopesticide, within the same insect and pathogen, followed by the same small letter are not significantly different. Mean% mortality for different insect, within the same concentrations or biopesticide, followed by the same capital letter are not significantly different (Bonferroni test, a = 0.05). Estimate Median survival time (days) of the same insect followed by the same small letter are not significantly different (Kaplan–Meier, a = 0.05). * Median Survival Time (*S. zeamais*: F: 11.000; *d*_f_: 6; *p* = 0.225, *R dominica*: F: 1.565; *d*_f_:6; *p* = 0.975, *P. truncatus*: F: 1.259; *d*_f_: 6; *p* = 0.877, *T. granarium*: F: 1.240; *d*_f_: 6; *p* = 0.893) (*n* = 200).

Insect	Concentration (ppm)				Mortality		Median Survival Time (Days) *			
	CBD Oil	Azatin	Helicovex	Madex	(%)	Sd	Estimate	Sd	95% Confidence Interval	
									Lower Bound	Upper Bound
*S. zeamais*	0	0	0	0	6	2.00	27.160a	0.510	26.160	28.160
	500	0	0	0	38bA	3.90	22.260b	1.251	19.809	24.711
	1500	0	0	0	54dA	1.40	21.140b	1.184	18.819	23.461
	3000	0	0	0	70eA	10.0	17.920c	1.274	15.424	20.416
	0	1500	0	0	46cA	1.40	23.800b	0.950	21.939	25.661
	0	0	1500	0	16aA	3.90	26.180a	0.787	24.638	27.722
	0	0	0	1500	18aA	3.40	26.180a	0.690	24.827	27.533
*R. dominica*	0	0	0	0	6	2.00	27.300a	0.4	26.500	28.100
	500	0	0	0	44bA	5.20	21.280b	1.266	18.798	23.762
	1500	0	0	0	60cA	2.20	20.720b	1.155	18.455	22.985
	3000	0	0	0	84dA	5.50	15.380c	1.228	13.272	18.088
	0	1500	0	0	44bA	1.40	23.240b	1.062	21.159	25.321
	0	0	1500	0	20aA	5.10	25.480b	0.904	23.708	27.252
	0	0	0	1500	22aA	4.50	25.620b	0.794	24.064	27.176
*P. truncatus*	0	0	0	0	6	3.00	27.020a	0.560	25.922	28.118
	500	0	0	0	48bA	3.50	21.140b	1.183	18.821	23.459
	1500	0	0	0	58bA	4.80	20.720b	1.262	18.246	23.194
	3000	0	0	0	80cA	10.0	15.920c	1.229	13.871	18.689
	0	1500	0	0	50bA	4.20	22.960b	1.093	20.817	25.103
	0	0	1500	0	20aA	7.10	25.200a	0.979	23.281	27.119
	0	0	0	1500	20aA	7.10	26.180a	0.722	24.766	27.594
*T. granarium*	0	0	0	0	8	2.00	27.020a	0.626	25.792	28.248
	500	0	0	0	44aA	5.20	21.700b	1.253	19.244	24.156
	1500	0	0	0	54bA	2.20	20.700b	1.163	18.420	22.980
	3000	0	0	0	62cA	2.30	19.600c	1.224	17.201	21.999
	0	1500	0	0	38aA	4.50	23.800b	0.953	21.933	25.667
	0	0	1500	0	20aA	7.10	25.620a	0.876	23.903	27.337
	0	0	0	1500	24aA	5.50	26.740a	0.537	25.687	27.793

**Table 2:** Mean% mortality and median survival time (days) adults of *S. zeamais*, *R. dominica*, *P. truncatus* and *T. granarium* treated with CBD oil in combination with Azatin, Helicovex and Madex. Mean% mortality for different concentrations or biopesticide, within the same insect and pathogen, followed by the same small letter are not significantly different; mean% mortality for different insect, within the same concentrations or biopesticide, followed by the same capital letter are not significantly different (Bonferroni test, a = 0.05). Estimate median survival time (days) of the same insect followed by the same small letter are not significantly different (Kaplan–Meier, a = 0.05). * Median survival time (*S. zeamais*: F: 0.207; *d*_f_: 8; *p* = 0.936, *R dominica*: F: 0.565; *d*_f_: 8; *p* = 0.975, *P. truncatus*: F: 3.667; *d*_f_: 8; *p* = 0.230, *T. granarium*: F: 0.125; *d*_f_: 8; *p* = 0.975).

Insect	Combined Concentration (ppm)				Mortality		Median Survival Time (Days) *			
	CBD Oil	Azatin	Helicovex	Madex	(%)	Sd	Estimate	Sd	95% Confidence Interval	
									Lower Bound	Upper Bound
*S. zeamais*	500	1500	0	0	36aA	4.90	25.060a	0.793	23.506	26.614
	1500	1500	0	0	68cA	1.98	20.440b	1.041	18.399	22.481
	3000	1500	0	0	84dA	3.48	15.400c	1.235	12.979	17.821
	500	0	1500	0	30aA	5.00	24.360a	1.009	22.382	26.338
	1500	0	1500	0	46bA	5.48	23.080a	0.953	21.212	24.948
	3000	0	1500	0	62cA	1.37	21.000b	1.156	18.734	23.266
	500	0	0	1500	18aA	4.37	25.900a	0.727	24.475	27.325
	1500	0	0	1500	22aA	3.04	25.060a	0.954	23.191	26.929
	3000	0	0	1500	40aA	1.07	22.960b	1.095	20.814	25.106
*R dominica*	500	1500	0	0	50bA	10.0	22.680a	1.020	20.681	24.679
	1500	1500	0	0	80dA	5.80	18.060a	1.081	15.941	20.179
	3000	1500	0	0	90eB	1.07	14.140b	1.176	11.835	16.445
	500	0	1500	0	40bA	5.80	23.380a	1.087	21.250	25.510
	1500	0	1500	0	60cA	2.25	22.260a	1.079	20.145	24.375
	3000	0	1500	0	70cA	1.07	19.880a	1.195	17.539	22.221
	500	0	0	1500	32aB	4.40	23.520a	1.072	21.419	25.621
	1500	0	0	1500	42bB	2.30	22.820a	1.089	20.686	24.954
	3000	0	0	1500	60cB	4.14	21.000a	1.174	18.699	23.301
*P. truncatus*	500	1500	0	0	44dA	2.37	23.520a	1.008	21.544	25.496
	1500	1500	0	0	74fA	5.17	19.320a	1.072	17.218	21.422
	3000	1500	0	0	94gB	5.40	13.300b	1.119	11.107	15.493
	500	0	1500	0	38cA	2.37	23.520a	1.072	21.419	25.621
	1500	0	1500	0	54eB	1.40	22.960a	1.049	20.904	25.016
	3000	0	1500	0	70fA	3.07	26.765a	1.198	24.416	29.114
	500	0	0	1500	26aB	1.40	24.220a	1.055	22.152	26.288
	1500	0	0	1500	30bC	2.25	24.220a	1.029	22.203	26.237
	3000	0	0	1500	52eC	3.04	21.000a	1.177	18.692	23.308
*T. granarium*	500	1500	0	0	50bA	1.07	22.680a	1.061	20.601	24.759
	1500	1500	0	0	56bB	2.94	21.420a	1.183	19.102	23.738
	3000	1500	0	0	62cC	2.37	20.580a	1.213	18.203	22.957
	500	0	1500	0	46aA	3.48	22.820a	1.105	20.654	24.986
	1500	0	1500	0	54bA	3.40	22.260a	1.136	20.034	24.486
	3000	0	1500	0	68cA	2.37	20.440a	1.220	18.049	22.831
	500	0	0	1500	38aB	5.95	23.660a	1.074	21.554	25.766
	1500	0	0	1500	48aD	3.37	23.52a0	1.006	21.548	25.492
	3000	0	0	1500	54bC	3.48	22.680a	1.059	20.604	24.756

**Table 3:** Observed and expected mortality of *S. zeamais*, *R. dominica*, *P. truncatus* and *T. granarium* adults at the end of the experiment (28 days) treated with CBD oil in combinations with Azatin, Helicovex and Madex, plus their interaction (A = Additive. C = Competitive. S = Synergistic) (*n* = 200). * Expected mortality is calculated according to Robertson and Preisler [51].

Combined Concentration (ppm)				Mortality (%)		χ^2^ (1 *d*_f_; *p* = 0.05)	Interaction
CBD Oil	Azatin	Helicovex	Madex	Observed	Expected *		
*S. zeamais*							
500	1500	0	0	36	68	24.53	C
1500	1500	0	0	68	76	14.33	C
3000	1500	0	0	84	84	0.023	A
500	0	1500	0	30	51	8.864	C
1500	0	1500	0	46	64	7.512	C
3000	0	1500	0	62	76	6.224	C
500	0	0	1500	18	52	23.452	C
1500	0	0	1500	22	64	39.543	C
3000	0	0	1500	40	76	38.247	C
*R dominica*							
500	1500	0	0	50	70	10.129	C
1500	1500	0	0	80	78	0.033	A
3000	1500	0	0	90	91	0.161	A
500	0	1500	0	40	57	6.562	C
1500	0	1500	0	60	69	2.339	A
3000	0	1500	0	70	87	15.251	C
500	0	0	1500	32	58	14.995	C
1500	0	0	1500	42	70	19.831	C
3000	0	0	1500	60	88	38.586	C
*P. truncatus*							
500	1500	0	0	44	75	26.968	C
1500	1500	0	0	74	80	1.236	A
3000	1500	0	0	94	90	0.678	A
500	0	1500	0	38	60	11.007	C
1500	0	1500	0	54	68	4.808	C
3000	0	1500	0	70	84	8.757	C
500	0	0	1500	26	60	25.568	C
1500	0	0	1500	30	68	34.148	C
3000	0	0	1500	52	85	42.509	C
*T. granarium*							
500	1500	0	0	50	68	7.499	C
1500	1500	0	0	56	73	8.150	C
3000	1500	0	0	62	78	3.139	A
500	0	1500	0	46	58	3.372	A
1500	0	1500	0	54	66	3.292	A
3000	0	1500	0	68	72	0.403	A
500	0	0	1500	38	60	10.952	C
1500	0	0	1500	48	67	9.017	C
3000	0	0	1500	54	73	9.675	C

**Table 4:** Toxicity variables in the equation from Cox regression for the treatments against *S. zeamais*, *R. dominica*, *P. truncatus* and *T. granarium* adults at the end of Table. 28 days. ^†^ B: B values are associated with increased hazard and decreased survival time; as the predictor increases, the hazard of the event increases and the predicted survival duration decreases. Negative coefficients indicate decreased hazard and increased survival times. ^††^ Exp (B): the ratio of hazard rates.

Variables in the Equation						
Combined Concentration (ppm)	B ^†^	Sd	Sig.	Exp (B) ^††^	95.0% CI for Exp (B)	
					Lower	Upper
CBD 500–Madex 1500	−0.354	0.167	0.034	0.702	0.506	0.973
CBD 1500–Madex 1500	−0.217	0.160	0.176	0.805	0.588	1.102
CBD 3000–Madex 1500	0.254	0.146	0.082	1.289	0.968	1.716
CBD 500–Helicovex 1500	−0.113	0.157	0.471	0.893	0.657	1.215
CBD 1500–Helicovex 1500	0.235	0.145	0.106	1.265	0.951	1.681
CBD 3000–Helicovex 1500	0.593	0.138	0.000	1.810	1.381	2.373
CBD 500–Azatin 1500	0.055	0.150	0.713	1.057	0.787	1.419
CBD 1500–Azatin 1500	0.670	0.137	0.000	1.955	1.495	2.556
CBD 3000–Azatin 1500	1.088	0.133	0.000	2.969	2.288	3.853
**Separately Concentration** **(ppm)**						
CBD 500	0.082	0.153	0.007	1.085	0.804	1.465
CBD 1500	0.396	0.143	0.000	1.485	1.123	1.964
CBD 3000	0.858	0.136	0.000	2.359	1.807	3.080
Control	−1.964	0.288	0.830	0.140	0.080	0.247
Helicovex 1500	−0.906	0.195	0.410	0.404	0.276	0.592
Madex 1500	−0.928	0.215	0.592	0.396	0.259	0.603
Azatin 1500	−0.879	0.191	0.315	0.415	0.285	0.604
